# Addition of Venetoclax to Azacitidine Did Not Improve Survival in Acute Myeloid Leukemia and Was Not Well Tolerated: Real World Experience

**DOI:** 10.3390/cancers18050841

**Published:** 2026-03-05

**Authors:** David Yanni, Nupur Krishnan, Rouslan Kotchetkov

**Affiliations:** 1 School of Medicine, University of Lancashire, Preston PR1 2HE, UK; dyanni@lancashire.ac.uk; 2 Michael G. DeGroote School of Medicine, McMaster University, Hamilton, ON L8P 1H6, Canada; nupur.krishnan@medportal.ca; 3 Hudson Regional Cancer Program, Royal Victoria Regional Health Centre, Barrie, ON L4M 6M2, Canada

**Keywords:** azacitidine, venetoclax, acute myeloid leukemia, efficacy, safety, real world

## Abstract

**Introduction**: Front-line therapy with Azacitidine (AZA) + Venetoclax (Ven) improved overall survival (OS) and remissions in acute myeloid leukemia (AML) patients ineligible for standard induction. Less is known about the outcome of AML treated with AZA + Ven in the “real world”. **Methods**: We assessed the comparative pattern of administration, tolerability, efficacy and safety of AZA vs. AZA + Ven administered at our cancer centre. We retrospectively reviewed all patients treated with AZA alone or AZA + Ven. Patients who received less than one cycle or proceeded with consolidative stem cell transplant were excluded. **Results**: A total of 53 patients, median age 77 years, received AZA, and 23 patients, median age 73 years, received AZA + Ven. Among those, 69% and 47.8% were ≥75 years old, respectively. Only 52% received Ven doses above 200 mg. Mean time on therapy was 13.1 months in AZA vs. 5.9 months in AZA + Ven. Treatment delays occurred in 22.6% of AZA and 34.8% of AZA + Ven patients, primarily due to infections and cytopenias. Neutropenia grade 3/4 occurred in 28.3% of AZA vs. 56.5% of AZA + Ven patients. Thrombocytopenia grade 3/4 occurred in 15.1% of AZA and 51.2% of AZA + Ven patients. Anemia grade 3/4 occurred in 5.7% of AZA vs. 30.4% of AZA + Ven patients. Moreover, 69.8% of AZA and 69.5% AZA + Ven patients reached stable disease/partial and complete remission. Median overall survival (OS) was similar: 18 months in AZA vs. 14 months in the AZA + Ven group, *p* = 0.905. **Conclusions**: In a community setting, the addition of Venetoclax to AZA did not improve overall survival or disease control, mainly due to low tolerability and higher toxicity. However, these results should be interpreted cautiously due to a significant imbalance in the cytogenetic risk profiles and lower tolerability in the combined group. This suggests the need for a larger study with adjusted analyses.

## Simple Summary

We compared the safety and efficacy of front-line therapy with azacytidine (AZA) alone or in combination with venetoclax (AZA + Ven) in AML patients who were not eligible for induction chemotherapy. AZA was well-tolerated, while only 52% received Ven doses above 200 mg. More patients on AZA + Ven had treatment delays. Hematological adverse events were more common in AZA + Ven groups. There were no differences in response rate and overall survival between AZA and AZA + Ven cohorts. We concluded that in the real-world setting, the addition of Venetoclax to AZA did not improve overall survival or disease control, mainly due to low tolerability and higher toxicity.

## 1. Introduction

Acute myeloid leukemia (AML) is an aggressive hematologic malignancy with a poor prognosis, especially among older adults, who represent the majority of cases and are not eligible for induction chemotherapy (IC). Outcomes are particularly poor for elderly patients due to unfavourable prognostic factors such as adverse cytogenetics, poor performance status, and comorbidities [1]. Patients aged ≥65 years with AML have a median overall survival (OS) of only 2–8 months. Best supportive care alone is associated with the lowest survival rates (median OS: 2 months), while low-dose cytarabine has been shown to slightly improve outcomes (median OS: 5 months). However, neither approach is universally accepted, and there remains no clear consensus on optimal therapy for this vulnerable population.

Hypomethylating agents such as azacitidine (AZA) and decitabine have emerged as viable options for treating AML patients ineligible for induction therapy. Azacitidine prolonged survival when compared with conventional care regimens (CCRs) in the subset of older patients with bone marrow blasts between 20% and 30% in the AZA-001, a multicenter, randomized, open-label, phase 3 trial [2]. AZA improved median overall survival: 10.4 months vs. 6.5 months and one-year survival rates: 46.5% vs. 34.2% compared to CCRs. AML patients who participated in the Austrian Azacitidine Registry had Improvement of overall survival of 9 to 10 months [3]. While these outcomes were less robust than those observed in MDS, they established AZA as a new standard of care for older newly diagnosed AML patients with high blast counts [4].

Building on AZA’s success, efforts then focused on enhancing its efficacy through combination therapies. Venetoclax (Ven), a selective BCL-2 inhibitor, has shown preclinical synergy with AZA by inducing apoptosis in AML cells. The VIALE-A, a phase III randomized controlled study, was a pivotal trial in which 286 patients received AZA + Ven, and 145 patients received placebo + AZA. Venetoclax was given orally at 400 mg daily, on a 28-day cycle, and AZA was administered to all patients at the standard dose: 75 mg/m^2^ subcutaneously or intravenously on days 1 through 7 every 28-day cycle. This study showed that the addition of Ven to AZA in treatment-naïve AML patients ineligible for IC further improved overall survival to 14.7 months compared to 9.6 months of AZA alone. It showed an improved composite complete remission rate (66.4%) compared to AZA alone (28.3%) [5]. These results established AZA + Ven as a new standard of care for this challenging population, albeit with increased toxicity, including higher rates of grade 3/4 neutropenia (42%) and febrile neutropenia (30%).

Despite promising trial outcomes, first real-world experiences with AZA and AZA + Ven showed different outcomes compared to phase 1b/II and phase III clinical trials [6]. Introducing AZA + Ven into the real-world setting (RWS) led to significant practice variation, mainly due to low tolerability in patients as compared to clinical trial populations. We therefore assessed the efficacy, tolerability, safety and pattern of administration of AZA + Ven and compared it to AZA alone in non-selected AML patients who were not fit or not eligible for IC.

## 2. Materials and Methods

### 2.1. Patient Selection

We conducted a retrospective review of all patients diagnosed with AML at the Hudson Regional Cancer Program, Barrie, Ontario, Canada, who received treatment with either AZA monotherapy or a combination of AZA + Ven. Patients who received at least one cycle of their respective treatment regimens were included in the analysis. Patients who received oral azacitidine or those who proceeded to consolidative allogeneic stem cell transplantation were excluded. AZA was administered subcutaneously at 75 mg/m^2^, Ven was given orally, starting dose of 100 mg daily on Days 1 and 2, then 200 mg starting on Day 3 and aiming to increase to 400 mg daily starting Day 4 and onwards. Patient demographics and baseline characteristics were collected, including age and the proportion of patients aged ≥75 years. All patients underwent standard pre-treatment work-up, including a physical examination, complete blood count, serum chemistry, bone marrow examination and other investigations as needed. Pharmacy performed the patient’s medication review as a standard of care to minimize drug interactions and enhance compliance.

### 2.2. Efficacy and Safety Assessment

Efficacy was assessed by overall survival (OS), (primary endpoint), and rates of response (secondary endpoint), including complete remission (CR), complete remission with incomplete recovery (CRi), partial remission (PR), and stable disease (SD). CR was defined as an absolute neutrophil count of more than 1000 cells per cubic millimetre, platelet counts of more than 100,000 per cubic millimetre, red-cell transfusion independence, and/or bone marrow with less than 5% blasts. Cri was defined as <5% blasts in bone marrow and absolute neutrophil count (ANC) between 1000 and 1500/µL and/or platelets between 100,000 and 150,000/µL. PR was defined as all the criteria for complete remission, except that both the neutrophil and platelet counts were lower than the threshold designated for Cri (ANC between 500 and 1100/µL and a platelet count between 50,000 and 100,000/µL).

Patients who did not meet PR criteria but did not progress were considered stable disease (SD) [7]. Progressive disease was defined according to the recommendations of the European Leukemia Net [8]. Cytogenetic risk was evaluated by the investigators according to the NCCN guidelines for AML, version 2.2024. Transfusion independence was defined as the absence of a red cell or platelet transfusion for at least 56 days at any point. Survival outcomes were assessed using Kaplan–Meier survival curves for each treatment group. OS was defined as the number of days from the start date of AML therapy to the date of death [9]. Disease-free survival (DFS) was measured from the time AML therapy started until disease recurrence [10,11]. Data for each patient were censored at the date of the last visit or the date on which the patient was last known to be alive. Where possible, disease assessments were performed with the use of the modified International Working Group response criteria for AML [12]. Toxicity was assessed as described previously [13].

Safety was assessed by collecting the hematological and non-hematological side effects, hospitalizations, and using the clinical notes and electronic medical records. Rates of adverse events (AEs) and tolerability were secondary study endpoints. AEs were defined as those that occurred from the first dose until 30 days after the discontinuation of treatment. AEs, defined as any untoward medical occurrence that may present during treatment with a pharmaceutical product, but which does not necessarily have a causal relationship with this treatment [14], were collected at each therapy cycle during routine clinical assessment, reported by patients directly, or by other health care providers. In addition, patients were assessed at the Toxicity Assessment Clinic at our cancer centre. The severity of AEs was graded according to the National Cancer Institute Common Terminology Criteria for Adverse Events, version 4.03 [15].

### 2.3. Statistical Analysis

We assessed demographic and AML disease characteristics (including cytogenetic and molecular), doses and schedule of AZA and Ven; and safety (including acute and late toxic effects). We assessed the rates of overall and complete responses after each therapy cycle. On the cut-off date for this analysis (2 January 2025), we censored data for patients who had no reported events at the most recent assessment. We used the Kaplan–Meier method to estimate survival curves and applied the log-rank test for the comparisons. ECOG levels were categorized as 0 or 1 vs. 2 or 3 based on clinical expertise. Statistical analyses were conducted to compare OS, DFS and tolerance between the two regimens. Prism 10.1 software was used for statistical analysis.

## 3. Results

### 3.1. Patient Characteristics

**Figure 1:**
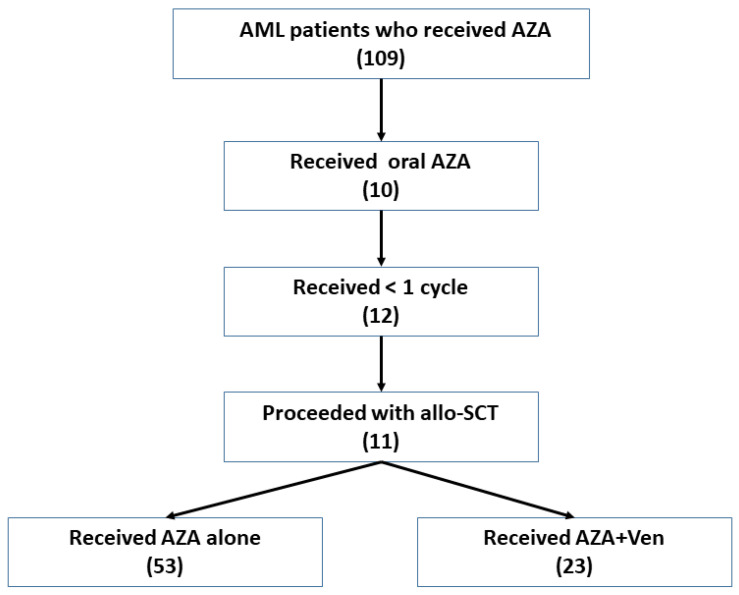
Patient disposition showing patient preselection.

A total of 109 patients with AML received AZA. In total, 12 patients received less than 1 cycle of AZA, 11 patients proceeded with allo-SCT, and 10 patients received oral AZA. Those patients were excluded from the analysis. A total of 76 patients who fulfilled the inclusion criteria were identified, with 53 receiving palliative AZA monotherapy and 23 receiving AZA + Ven (Figure 1).

Decision for each treatment was made by a treating hematologist based on availability and funding of the therapy regimen, patient’s preference, and estimated ability to tolerate therapy. Cytogenetics and molecular tests were not available at the time of treatment allocation.

Baseline characteristics are shown in Table 1. Among the AZA cohort, patients had a median age of 77 years (range: 59–92), with 71.7% being ≥75 years old. In the AZA + Ven cohort, patients’ median age was 73 years (range: 54–83), with 56.5% being ≥75 years old. Secondary AML included 21 patients (39.6%) in the AZA group vs. 6 patients (26.1%) in the AZA + Ven cohort (*p* = 0.305). Comorbidities were equally prevalent in both groups, with a median of 4 comorbid conditions per patient (range: AZA 0–11, AZA + Ven 0–17). Secondary cancers, such as breast cancer and chronic lymphocytic leukemia, were infrequent, affecting 3.5% of AZA patients and 4.3% of AZA + Ven patients. High cytogenetic and molecular risk profiles were more frequent in the AZA + Ven group (65.2%) compared to the AZA group (32%), *p* = 0.011. Median ECOG performance status was 2 in the AZA group and 1 in the AZA + Ven group. ECOG 3 had 20.8% of patients in AZA % and 21.3% of patients in the AZA + Ven cohorts (*p* = 0.991). There was no difference in the number of comorbidities, median blast count and transfusion dependency at baseline between groups as shown in Table 1 below.

Azacitidine was delivered consistently in both groups, with 96% of AZA patients and 95.6% of AZA + Ven patients receiving full doses, 75 mg/m^2^ over 7 days per cycle (Table 2). Median dose of AZA was 75 mg/m^2^ in both groups; however, median number of AZA cycles delivered was higher in the AZA group: 6 vs. 5 in AZA + Ven. The difference was statistically significant, *p* = 0.049. However, the AZA + Ven cohort experienced significant challenges with venetoclax dosing. The median daily dose of venetoclax was 200 mg, with only 52% of patients tolerating doses ≥200 mg.

The mean duration of therapy was notably longer in the AZA group (13.1 months, range: 1.15–92.9) compared to the AZA + Ven group (5.9 months, range: 1–18), with a median of 14 cycles (AZA) versus 5 cycles (AZA + Ven). Treatment delays occurred less often and were shorter in AZA patients (12 (22.6%), median delay time of 14 days [range 7–28 days] compared to the AZA + Ven cohort (8 patients (34.8%), median delay time of 28 days, range [7–56 days]). Delay due to infections was the most common cause for both groups: 10 (18.9%) patients in AZA and 4 (17.4%) patients in AZA + Ven group, *p* = 0.697. Delays due to cytopenia were less frequent in the AZA cohort (2 patients (3.4%) compared to 8 patients (17.4%) in the AZA + Ven group, *p* = 0.034. Patients continued therapy until progression or intolerance.

### 3.2. Patient Safety

Hematologic adverse events were significantly more frequent in the AZA + Ven cohort, affecting 91.3% of patients compared to 43.4% in the AZA cohort, as shown in Table 3 below. Anemia was almost universal in the AZA + Ven group (91.3%) and less common in the AZA group (49.0%), with severe anemia (grade 3/4) affecting 30.4% and 7.4% of patients, respectively. Grade 3/4 neutropenia was reported in 56.5% of AZA + Ven patients versus 28.3% in the AZA group. Similarly, grade 3/4 thrombocytopenia affected 43.5% of AZA + Ven patients compared to 12.5% of AZA patients. Non-hematologic adverse events occurred more frequently in the AZA + Ven group as well. Infections, including febrile neutropenia, pneumonia, and sepsis, were the leading causes of morbidity.

Febrile neutropenia was more common in the AZA + Ven group (34.8%) compared to the AZA group (14.3%). Pneumonia and sepsis affected 18.4% and 9.2% of AZA patients, respectively, compared to 39.3% and 22.1% of AZA + Ven patients. In total, 45.5% of AZA + Ven patients required therapy with antibiotics, compared to 19.9% of AZA patients. Compared to AZA alone, patients in the AZA + Ven cohort required more platelets (49.8% vs. 18.5%) and red blood cell (28.8% vs. 7.1%) transfusions. Only one patient on Ven + AZA received G-CSF for a life-threatening infection. Gastrointestinal symptoms, including nausea and constipation, were similar in both groups but occurred more frequently in the AZA + Ven cohort.

A total of 49 patients in the AZA group and 14 in the AZA + Ven group died. Disease progression was the most common primary cause of death in both groups, with no difference: 14 AZA patients (28.6%) and 3 AZA + Ven (21.4%) patients. Febrile neutropenia, sepsis, and respiratory infections were more frequent in AZA + Ven groups. However, more patients in the AZA group decided on palliation and medical assistance in dying (Table 4).

### 3.3. Efficacy

**Figure 2:**
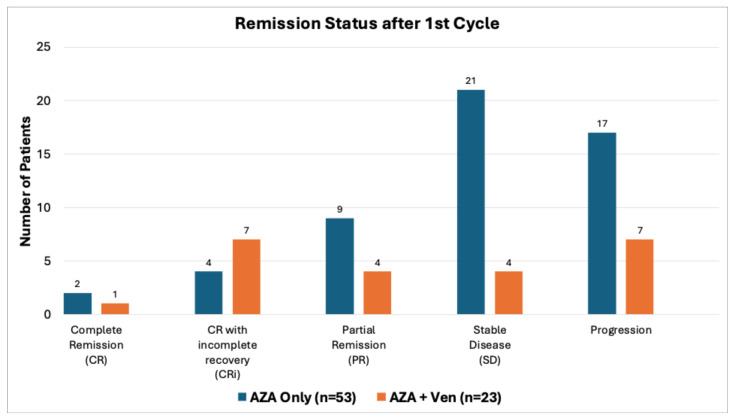
Remission status after cycle 1. The difference between rate of stable disease and rate of progression was statistically significant (*p* < 0.05).

**Figure 3:**
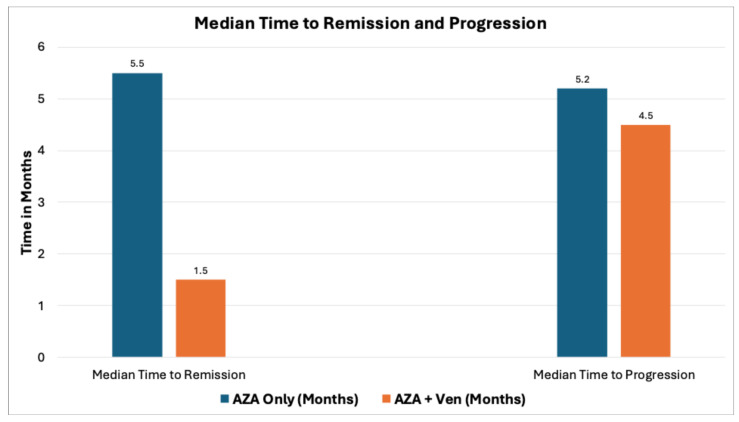
Time to remission vs. time to progression, The difference between median time to remission and median time to progression was statistically significant (*p* < 0.05).

Both groups showed similar overall response rates, with 69.8% of AZA patients achieving stable disease, partial remission, complete remission (CR), or CR with incomplete count recovery (CRi) compared to 69.5% in the AZA + Ven group. However, only a small number of patients reached CR in both groups. The CRi rate was higher in the AZA + Ven group (30.4%) compared to AZA monotherapy (7.5%), *p* = 0.002, Table 2. Disease progression occurred in 32% of AZA and 30.4% of AZA + Ven patients, with no statistical difference between the groups. Transfusion independence reached 52.8% of patients in AZA and 65.2% in the AZA + Ven cohort. None of the AZA patients reached complete remission after cycle 2; significantly fewer AZA + Ven patients had progression after cycle #1. On the other hand, more patients in the AZA group had stable disease after cycle 1, as shown in Figure 2. Median time to remission was longer in AZA (5.5 months) vs. 1.5 months in AZA + Ven group (*p* = 0.002). However, there was no difference in median time to progression between AZA and AZA + Ven groups (Figure 3).

**Figure 4:**
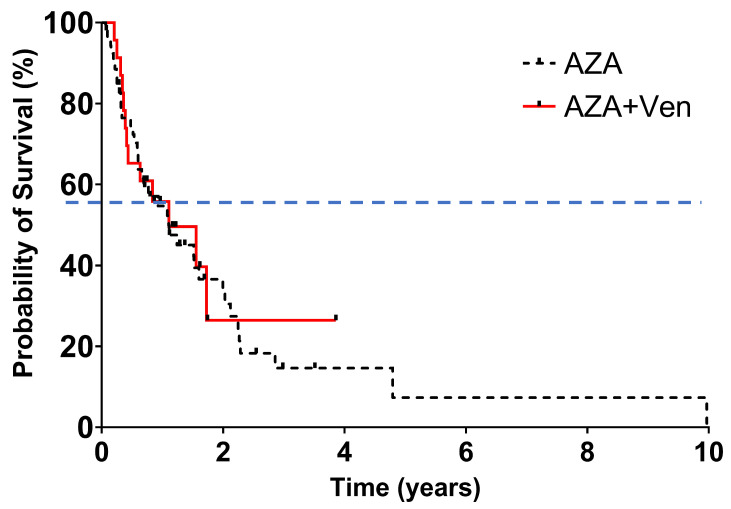
Overall survival. Median follow-up 12.3 months, hazard ratio 1.065 (95% CI 0.56–2.01), *p* = 0.8134. The distributions were estimated for each treatment group with the use of the Kaplan–Meier method and were compared with the log-rank test. The hazard ratio for death was estimated with the use of the Cox proportional-hazards model. The data included are subject to a cut-off date of 2 January 2025. The dashed line indicates 50% overall survival probability, and the tick marks indicate censored data.

**Figure 5:**
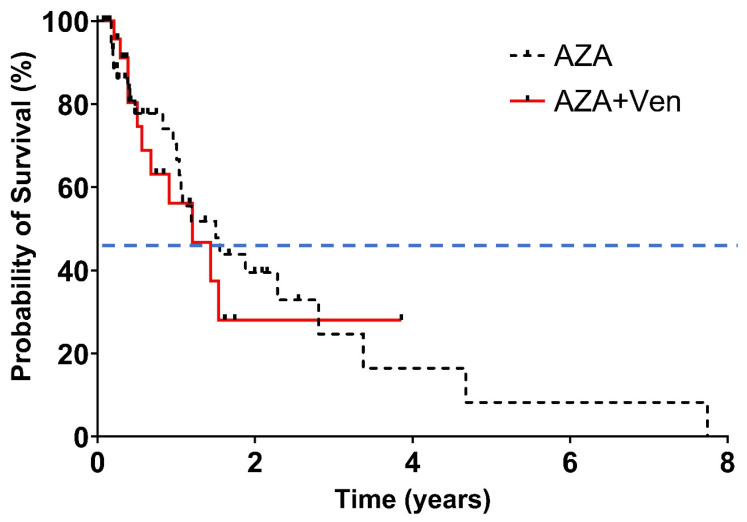
Disease-free survival. Median follow-up 21.3 months, HR (0.875, 95% CI 0.413–1.854), *p* = 0.905. The distributions were estimated for each treatment group with the use of the Kaplan–Meier method and were compared with the log-rank test. The hazard ratio for death was estimated with the use of the Cox proportional-hazards model. The data included are subject to a cutoff date of 2 January 2025. The dashed line indicates 50% overall survival probability, and the tick marks indicate censored data.

Despite these response differences, it did not translate to a statistically significant difference in median OS: 18 months in the AZA group versus 14 months in the AZA + Ven group (*p* = 0.905), as shown in Figure 4. Disease progression and infections were the leading causes of death, accounting for 30.2% and 26.4% of fatalities in the AZA group, and 26.1% and 30.4% in the AZA + Ven group, respectively. DFS was very similar to OS, showing no significant differences (Figure 5).

## 4. Discussion

Even though AZA + Ven showed improved survival and disease control compared to AZA monotherapy and was established as a standard of care for newly diagnosed AML patients not eligible for IC, its efficacy and safety in a real-world setting are more challenging to maintain when compared to clinical trials. This reflects the real-world challenge of managing AML in frail patients who are ineligible for intensive induction chemotherapy. Clinical trial populations are often younger, less comorbid, and more closely monitored compared to the heterogeneous and frail patients seen in community oncology settings. Real-world AZA monotherapy studies, such as those conducted by the Austrian Azacitidine Registry and French Compassionate Use Program, have reported a median OS of 9–10 months for AZA monotherapy in AML [14]. These findings align with the AZA-AML-001 trial but fall short of the survival benefits observed in MDS populations.

The addition of venetoclax introduces further challenges in real-world settings. Dose adjustments, treatment delays, and interruptions due to hematologic toxicity are common, limiting the applicability of trial protocols. Moreover, supportive care measures, such as prophylactic antibiotics and growth factor administration, are less rigorously implemented in non-trial settings, contributing to poorer outcomes. The results of AZA-VEN application in the real world, outside of phase 1b/II and phase III clinical trials, are rather disappointing. For example, the median duration of AZA-VEN exposure in the real-world ranges between 3.6 and 7 months, compared to 7 months in VIALE-A [6]. The median OS for AZA-VEN in RWS varies from 9.2 to 12.7 months, compared to 14.7 months in VIALE-A. Biochemical tumour lysis syndrome (TLS) in RWS is reported to be as high as 18%, compared to only 1% in VIALE-A (5). We showed a high prevalence of patients with poor ECOG status, indicating substantial functional impairment. Comorbidities were prevalent in both groups, with a median of four comorbid conditions per patient; however, some patients had up to 17 conditions. The high burden of comorbidities underscores the complexity of treatment in this population, where overlapping health issues may exacerbate toxicity and compromise therapy adherence. The difference in adverse cytogenetics and molecular risk profile may contribute to an imbalance between the groups, with AZA + Ven patients having more aggressive disease, aligning with its role as an intensified treatment regimen. Based on our early experience, high-risk patients may require more vigilant follow-up and more intensified supportive therapy while on AZA + Ven therapy.

In addition, the significantly higher rates of hematologic toxicity in the AZA + Ven cohort raise concerns about the balance between efficacy and safety. While the combination achieved higher CR rates, the associated toxicities often necessitated treatment interruptions, potentially diminishing its clinical benefit. We found that our real-world patient population was able to receive several Ven cycles comparable to those in clinical trials. Successful delivery of AZA-VEN is an acquired art [6], and proactive monitoring and early intervention for cytopenias and infections are critical to minimizing morbidity and optimizing outcomes. That emphasizes the importance of tailoring treatment strategies for frail AML patients. The challenges in venetoclax dose escalation and the shorter treatment duration in the AZA + Ven group highlight the difficulties of implementing trial-based protocols in real-world settings. In our cohort, only half of the patients could tolerate doses ≥200 mg. The target dose of 400 mg, as recommended in the VIALE-A trial, was achieved in just a fraction of patients. Dose reductions and delays due to febrile neutropenia and cytopenias reduce the intensity of therapy and may negate the potential benefits of combination treatment. Ensuring optimal supportive care could improve tolerability and allow more patients to achieve target dosing. We had to modify the AZA + Ven therapy protocol in patients based on their tolerability of the first cycle by reducing the number of Ven cycles to 1 and decreasing the treatment duration from 28 to 21 days or even 14 days to improve tolerability and increase time on treatment.

Our study has several limitations: The number of patients is small, and thus there is no significant statistical power to detect true differences, particularly for subgroup analyses and overall survival. Due to the nature of the real-world setting and retrospective nature of the study, we could not balance baseline characteristics between AZA and AZA + Ven groups. In addition, the absence of adjusted multivariable analyses means we cannot fully account for the confounding effect of the significant imbalance in cytogenetic and molecular risk profiles between the two groups. Therefore, the clinical recommendations we make cannot be generalized, and they reflect our local experience only. Next, in the real-world setting, the intervention was not delivered as designed in clinical trials, with only half of the patients receiving the planned Ven dose of 400 mg. Thus, delivery of Ven + AZA in the real world deviated substantially from a clinical trial protocol. In addition, we were not able to properly assess minimal residual disease in the community setting.

The AZA group’s longer treatment duration and lower toxicity suggest that monotherapy may still be suitable for patients with functional impairments or extensive comorbidities, who are less likely to tolerate intensified regimens. The similar OS in both groups suggests that AZA monotherapy provides comparable survival benefits in real-world settings, despite the higher CR rates observed with AZA + Ven. This raises questions about whether higher CR rates are clinically meaningful if they do not translate to an improved OS.

This aligns with findings from other real-world studies, which have shown that AZA remains a valuable treatment option for older AML patients, particularly those with less aggressive disease or limited supportive care access. The results of this study provide valuable insights into the challenges and opportunities of treating AML in real-world settings. While AZA + Ven has demonstrated clear efficacy in clinical trials, its translation into routine practice remains complex. Understanding the factors that influence outcomes—such as patient selection, supportive care, and toxicity management—will be crucial for refining treatment algorithms and improving survival for this vulnerable population. There is also a need for resource allocation and further testing to refine patient selection for AZA + Ven therapy. While the combination of AZA + Ven offers higher response rates in certain subsets of patients, the significant toxicity associated with this regimen limits its suitability for frail populations or those with extensive comorbidities. To address these challenges, targeted research is essential to develop tools and strategies that identify patients who are most likely to benefit from AZA + Ven therapy while minimizing harm. Expanding the study to include a larger cohort and conducting prospective analyses could provide more definitive insights into the comparative effectiveness of these regimens.

## 5. Conclusions

Based on our single-centre first experience, the addition of Venetoclax to AZA did not improve overall survival or disease control in patients not eligible for induction therapy. This lack of benefit may be influenced by the higher prevalence of adverse cytogenetic risks and the challenges of managing increased toxicity in a community setting. We suggest low tolerability and higher toxicity of our patients to the AZA + Ven combination. While AZA + Ven showed promising outcomes in the real world in terms of remission rates, especially after the first cycle, its low tolerability in subsequent cycles is a significant limiting factor. Potential reasons for the eventual progression of AZA + Ven patients include significantly higher susceptibility to hematological adverse effects, infections and limited support. These findings are suitable for generation of hypotheses and highlight the intricacies of translating clinical trial success into routine practice in the real-world. Further research is required on a larger population to verify the potential of AZA + Ven.

## Figures and Tables

**Table 1:** Patient demographics.

Patient Demographics	AZA, N = 53	AZA + Ven, N = 23	*p* Value
Median age at diagnosis, years [range]	77 [59–92]	73 [54–83]	0.049 *
<75 (%)	15 (28.3)	10 (43.5)	0.288
>75 (%)	38 (71.7)	13 (56.5)	0.287
Sex M/F	30/23	18/5	0.007
Primary AML (%)	32 (60.4)	17 (73.9)	0.331
Secondary AML (%)	21 (39.6)	6 (26.1)	0.305
Median number of comorbidities	4 [0–15]	4 [0–17]	0.881
Concomitant malignancies (non-hematological)	2 (3.5)	1 (4.3)	0.873
High risk cytogenetics/molecular (%)	17 (32.1)	15 (65.2)	0.011 *
Intermediate (%)	7 (13.2)	3 (13.1)	0.981
Favourable (%)	26 (49.1)	5 (21.7)	0.026 *
Unknown (%)	3 (5.6)	0	0.890
ECOG (%)			
0	4 (7.5)	3 (13)	0.578
1	22 (41.5)	12 (52.2)	0.024 *
2	16 (30.2)	3 (13)	0.043 *
3	11 (20.8)	5 (21.8)	0.991
4	0 (0)	0 (0)	N/A
Median % BM Blasts at Dx	28% [5–91]	30% [4–92]	0.531
Transfusion dependent (%)	28 (52.8)	14 (60.7)	0.618
Average number of comorbidities	6 [1–14]	5 [2–12]	0.698

Data presented as a number (N) and percentage (%); abbreviations: AZA, azacitidine, Ven, venetoclax, ECOG, * statistically significant.

**Table 2:** Treatment characteristics & response.

Treatment Characteristics	AZA Only, N = 53	AZA + Ven, N = 23	*p* Value
Median AZA dose, mg/m^2^ [range]	75 [64–75]	75 [60–75]	
Median Ven dose, mg [range]	N/A	200 [50–400]	N/A
Median N of AZA cycles [range]	6 [2–101]	5 [2–17]	0.049 *
Median N of Ven cycles [range]	N/A	5 [2–17]	N/A
Time on therapy, months, [range]	13.1 [1.15–92.9]	5.9 [1–18]	0.006 *
Treatment Delays, n (%)	12 (22.6)	8 (34.8)	0.395
Median delay time, days, [range]	14 [7–28]	28 [7–56]	0.035 *
Best Response, N (%)			
Complete remission	2 (3.8)	1 (3.8)	0.002 *
Complete remission with incomplete count recovery	4 (7.5)	7 (30.4)	0.002 *
Partial remission	9 (17.0)	4 (17.4)	0.994
Stable disease	21 (39.6)	4 (17.4)	0.008 *
Progression	17 (32.1)	7 (30.4)	0.731
Transfusion independency, N (%)	28 (52.8%)	14 (60.9)	0.618

Data presented as a number (N) and percentage (%); abbreviations: AZA, azacitidine, Ven, venetoclax; * statistically significant.

**Table 3:** Hematological and non-hematological AEs.

Adverse Event	AZA, N = 56	AZA + Ven, N = 23	*p* Value
Anemia	27, (49.0)	21, (91.3)	0.031 *
Grade 1 and 2	25 (92.6)	14 (60.9)	0.043 *
Grade 3 and 4	2, (7.4)	7 (30.4)	0.001 *
Thrombocytopenia	16, (8.9)	19, (82.6)	<0.001 *
Grade 1 and 2	14, (87.5)	9, (39.1)	<0.001 *
Grade 3 and 4	2, (12.5)	10, (43.5)	0.022 *
Neutropenia	19, (33.9)	18 (78.2)	<0.001 *
Grade 1 and 2	3, (5.4)	5, (21.7)	0.065
Grade 3 and 4	16, (28.3)	13, (56.5)	0.033 *
Febrile neutropenia	8, (14.3)	8, (34.8)	0.003 *
Pneumonia	10, (18.4)	9, (39.3)	0.028 *
Sepsis	5, (9.2)	5, (22.1)	0.012 *
Nausea	19, (34.2)	14 (60.9)	0.009 *

Data presented as a number (N) and percentage (%); abbreviations: AZA, azacitidine, Ven, venetoclax; * statistically significant.

**Table 4:** Cause of death.

Primary Cause, N (%)	AZA, N = 49	AZA + Ven, N = 14	*p* Value
AML progression	14 (28.6)	3 (21.4)	0.462
Febrile neutropenia/sepsis	12 (24.5)	5 (35.7)	0.073
Bleeding	1 (2.0)	2 (14.3)	0.043 *
Respiratory infections	4 (8.2)	4 (28.6)	0.005 *
Patient decision: palliation/MAID	9 (13.4)	0	-
Other **	3 (6.1)	0	-
Unknown	6 (12.2)	0	-

Data presented as a number (N) and percentage (%); abbreviations: MAID, medical assistance in dying; * statistically significant, *p* < 0.05; ** other causes include end-stage COPD, congestive heart failure, myocardial infarction.

## Data Availability

Data sets used in the analysis are available from the corresponding author upon reasonable request.

## Abbreviations

The following abbreviations are used in this manuscript:

AZA	Azacitidine
Ven	Venetoclax
ECOG	Eastern Cooperative Oncology Group
CR	Complete Remission
CRi	Complete Remission with incomplete count recovery
PR	Partial Remission
SD	Stable Disease
DFS	Disease-Free Survival
OS	Overall Survival
MAID	Medical Assistance in Dying
IC	Induction Chemotherapy
CCR	Conventional Care Regimens
AML	Acute Myeloid Leukemia
AE	Adverse Event
TLS	Tumour Lysis Syndrome
